# The challenges, opportunities and strategies of engaging young people who use drugs in harm reduction: insights from young people with lived and living experience

**DOI:** 10.1186/s12954-022-00663-z

**Published:** 2022-07-26

**Authors:** M-J Stowe, Orsi Feher, Beatrix Vas, Sangeet Kayastha, Alissa Greer

**Affiliations:** 1 Department of Family Medicine, School of Medicine, Faculty of Health Sciences, University of Pretoria, Pretoria, South Africa; 2 South African Network of People Who Use Drugs (SANPUD), Cape Town, Western Cape South Africa; 3 PsyCare Austria, Vienna, Austria; 4 YouthRISE, Co Kerry, Ireland; 5 Unite-Global Parliamentarians Network to End Infectious Diseases, Lisbon, Portugal; 6 Y-PEER Asia Pacific Center, Bangkok, Thailand; 7 School of Criminology, Simon Fraser University, Burnaby, BC Canada

**Keywords:** Young people who use drugs, Youth; drug use, Harm reduction, Engagement, Lived-experience, Participation, Involvement

## Abstract

The meaningful inclusion of young people who use or have used drugs is a fundamental aspect of harm reduction, including in program design, research, service provision, and advocacy efforts. However, there are very few examples of meaningful and equitable engagement of young people who use drugs in harm reduction, globally. Youth continue to be excluded from harm reduction programming and policymaking; when they are included, they often face tokenistic efforts that lack clear expectations, equitable work conditions, and are rarely afforded agency and autonomy over decision-making. In this commentary, we identify and discuss issues in youth engagement, and offer recommendations for the future of harm reduction.

## Introduction

Globally, engaging people who use drugs (PWUD) from key populations, including people who inject drugs, women who use drugs, people living with HIV, transgender people and people who identify as LGBTQI+, is fundamental to harm reduction [1]. Grassroots organizing, along with “peer” engagement initiatives, have paved the way for the active participation of PWUD in harm reduction policy making, research, programming, advocacy, and service provision [2]. This inclusionary approach is characterized by a bottom-up, inclusive, and community-led approach to decision-making. It has the potential to facilitate equitable access to harm reduction services by promoting communication, developing trust and rapport, increasing knowledge, and reducing stigma and discrimination [3]. Despite the increasing support for engaging PWUD across various populations and countries [4], there is limited evidence and focus on the engagement of *young* PWUD in harm reduction [5].

In this commentary, we offer a much-needed perspective and discussion on the importance and value of youth engagement in harm reduction. We, the authors of this commentary, are young PWUD and allies from five countries. We draw on our lived-experiences, experiential knowledge and expertise to identify and discuss the key challenges, opportunities, and recommendations for the future of youth engagement in harm reduction.

### Youth and harm reduction

Youth is an elastic category; it encapsulates multiple age groups and refers to the various stages in a person's life when they are not children but not yet entirely autonomous adults [6]. It is a period of one’s life that might be thought of as minors under 18–19 years old, young adults up to age 35, or even soon after childhood (10–13 years of age). Different cultures mark the end and the beginning of this phase differently, as is recognized across the United Nations [7], but it is uniformly considered formative years, when certain aspects of life and issues are more relevant than others [8]. One’s identity and positioning in the world are formed during this time. People in this period develop greater independence and agency, which includes confidence in their abilities to independently think and make decisions.

Although the term “youth” is loosely defined across various literature and policy making, most researchers and practitioners agree that initiation into drug use typically occurs in the late teens but can occur at much younger or older ages [9]. The age that someone first uses drugs can have a major impact on their drug use trajectories and the harms experienced [8]. Studies show that initiating drug use at a younger age is predictive of developing health and social issues later on [6, 10].

Given the risks and potential harms associated with drug use at a younger age [6], it is concerning that many young PWUD experience stigma, discrimination and barriers to accessing health and harm reduction services due to their age [11]. For example, we have experienced age restrictions when trying to access basic healthcare services, such as HIV testing or counselling, and health workers who are reluctant to provide us with services, particularly opioid substitution treatment and other medication therapies. Our experiences are not isolated and are corroborated in the literature (e.g. [9]). Considering the importance and clear deficiencies in services for young PWUD, engaging youth from a multitude of intersecting identities and backgrounds in the development, implementation, and evaluation of services is imperative in the design of efficient, accessible services.

### The benefits of youth engagement in harm reduction

In our experiences of working in and alongside various not-for-profit organizations internationally, most young PWUD are practical, action-oriented and possess a diversity of skills. The next generation of PWUD are natural experts on the emerging trends in drug use behaviour [3], and can offer unique insights on the realities of drugs and their use, particularly because of their experiential knowledge, connection to the community, and access and literacy with new technologies [12]. Young PWUD are able to provide insights and access to the places and spaces where young people use drugs and provide essential harm reduction “where they’re at”. Young PWUD are already present in spaces occupied by youth in various physical environments and social contexts, including places like festivals, schools, and various groups or communities. In these environments, social media and other technologies and communication channels can be leveraged to disseminate information in real-time [12]. These characteristics and attributes equip young PWUD with experiences and expertise to effectively respond to the true needs of their peer groups.

Young PWUD are often at a higher risk for HIV and viral hepatitis infection, as well as higher rates of fatal and non-fatal overdoses in certain contexts [13, 14], while engagement across key populations face multiple and intersecting challenges due to their social positioning [15]. Yet, reaching young people who inject drugs, live with HIV and/or hepatitis C, are sex workers, and who identify as a racial or ethnic minority and/or part of the LGBTQI+ community, is critical to effectively promoting access to healthcare services and evidence-based information. In our experience, this is most successfully done by well-prepared peers and by leveraging dynamic social networks [16, 17].

## Key challenges and issues

There are relatively few examples in the literature of harm reduction interventions that have effectively and meaningfully involved young PWUD as peer educators, mentors, programme designers, and evaluators while ensuring their agency and autonomy over decision-making processes. While some studies report encouraging outcomes on the inclusion of young PWUD [16, 18], most harm reduction interventions and programmes remain largely developed, studied, and funded to focus on adults, with little regard to the specific needs of younger PWUD [12].

Below, we offer our insight on several key issues related to youth engagement in harm reduction, including: (1) youth exclusion from harm reduction programming; (2) youth exclusion from key drug policies and reforms; (3) tokenism in youth engagement. Following this, we offer several recommendations to mitigate these issues.

### Youth exclusion from harm reduction programming

In our experiences, spaces that are meant for young PWUD, but are controlled by older peers, are an obstacle that can be addressed through youth engagement. A sense of top-down, age-based hierarchy can create aversion and resistance in young PWUD to both access and partake in services—thus, alienating the group that services are intended for.

The marginalization of youth can occur in several ways. For example, when adults use their authority to design and implement interventions that *they* deem appropriate for young people, such as school-based drug education programming, without any input from youth. Not engaging youth in the design and implementation process may result in decreased up-take of young PWUD and an increased stigma around seeking help [13]. Instead, youth engagement may empower young people by centring their voices in the design, decisions, delivery, and advocacy of harm reduction interventions and programmes.

Young PWUD are a diverse and heterogeneous population. While young PWUD face unique age-related barriers, lack of autonomy and agency over decision-making in harm reduction, this occurs within a broader societal context where youth are disproportionately affected by a number of socioeconomic, political, cultural and racial factors. Youth who face marginalization on the basis of racism, gender discrimination, poverty, ableism, and other positioning face overlapping barriers to services. Harm reduction programming for young people cannot be a one-size-fits-all solution that treats young PWUD as a homogenous group. It is imperative that inputs from young PWUD are centred in all its diversity in all aspects of harm reduction.

### Youth exclusion from drug policy reform

Drug policy reform is an important part of harm reduction. Currently, reigning drug policies that criminalize young PWUD are responsible for some of the most severe harms associated with drug use. The consequences of the prohibitionist approach include greater relative harms for young PWUD that can have a lasting impact long into adulthood. To be able to effectively craft policies that “leave no one behind” (as is the motto of the United Nations Office on Drugs and Crime) and initiatives that are “harnessing the transformative power of youth” [19], it is critical that youth and their lived experience are meaningfully included in drug policy reform activities, globally.

Young PWUD are at the forefront of advocating for progressive drug policy reform. Youth-led organisations are spearheading the global drug policy reform movement. Such organizations, often with minimal funding or support from “adult” peers, are promoting the voices, experiences, and expertise of young PWUD at both high-level government forums, as well as the important work of non-governmental committees.

However, young PWUD still face challenges and barriers to engaging in drug policy reform. While most policy makers justify their decisions with the important aim of protecting the youth, there is concerning evidence that young people are being excluded from both the decision-making process and policies themselves [12]. For example, in British Columbia, Canada, decriminalization efforts have not only excluded youth from the process, but policies do not apply to young people altogether, keeping young PWUD as “minors”, like alcohol and tobacco policies. In addition, the authors have personally experienced difficulties in engaging decision-makers and people in positions of decision-making when it comes to the design of international guidelines for service provision and youth inclusion. These challenges can range from national representatives actively ignoring individuals to blatant verbal abuse while our colleagues present their findings on the floor of the Commission on Narcotic Drugs at the UN. This exclusion of young PWUD is at the root of inefficient and, at times, harmful policies. Importantly, crafting drug laws and policies that are equitable and reflect the true needs and interests of young people requires youth engagement throughout the policymaking process.

### Tokenism in youth engagement

The marginalization of young PWUD can continue to occur when engagement efforts are tokenistic. Tokenism occurs when service providers selectively choose or “cherry-pick” youth representatives based on, for example, agreeability to deliver a pre-crafted message on behalf of other young PWUD. In our experiences, we have seen examples of tokenism in programmes where young PWUD are recruited to advise on content or deliver services that they had no meaningful engagement in—where they did not have the opportunity to shape or influence the initiatives. Tokenistic youth involvement only ticks the boxes of inclusivity and participation, but entails no meaningful engagement of young PWUD.

There is an assumption that young people can only be experts on “youth topics” and thus a tendency to limit involving young PWUD solely in initiatives or aspects of projects that are “youth-focused”—which tokenizes them for only *parts* of their identities. Initiatives often put youth in uncomfortable situations where they are expected to share personal details that might be sensitive in work or professional contexts, such as disclosing illicit or illegal acts. Further, the context and details of how their contributions will be used is sometimes withheld, for example, being asked to speak on behalf of an organization at an event, and being introduced as a young person who uses drugs when it was not discussed beforehand if they are comfortable disclosing that information.

In addition to limited agency and autonomy over decision-making, young PWUD are often only included in consultative roles, to provide comments and feedback on pieces of writing, projects or programmes, that have already been largely developed without their direct involvement. This severely limits the meaningful and transparent impact that young PWUD’s perspectives, often based on their lived experiences or expertise otherwise could have been gained.

PWUD, regardless of their age, possess a specific expertise and unique knowledge, the sharing of which should be compensated fairly—as with any other particular skill in any other field. When young people are solicited for work, they should be presented with transparent and accurate information about what is expected of them and be allowed space to negotiate the conditions in which they engage.

## Recommendations for the future of youth engagement in harm reduction

Despite the challenges, there are a number of opportunities to promote equitable and meaningful youth engagement in harm reduction. Kimmel et al. [12] developed principles of harm reduction for young people who use drugs, including that services are available and tailored towards youth. We echo these principles and underscore the value of an inclusive, meaningful, and equitable approach to harm reduction for youth. Such principles underpin our suggestions for the future of engaging youth in harm reduction. Here, we offer several recommendations to promote greater, meaningful engagement of young PWUD:

1. Ageist and exclusionary programming and processes in health and harm reduction programming must be urgently addressed—globally. Youth should not be excluded due to their age. Rather, this feature, along with other identities that youth are positioned in, is a valuable area of expertise for harm reduction.
2. The ability of young PWUD to self-organize and engage other youth as equal counterparts, building youth capacity and advocacy from the bottom up, must be incorporated into programme design and service delivery.
3. Harm reduction engagement efforts should draw on the leadership of autonomous youth-led organizations. Youth-led organizations allow for self-governing, self-determined agendas, and independent organization that prioritizes the interests and empowerment of youth above all else. Engaging youth through youth-led organizations can promote equity, diversity, and inclusion.
4. Youth engagement efforts must be informed by intersectionality and the lived-experiences of young PWUD across key populations, including those who identify as LGBTQI+ and racial and ethnic minorities. These efforts must not be tokenistic.
5. Young PWUD require pay and work equity in harm reduction. Learning from areas of research and advocacy, we know that young people are often expected to volunteer their time, work in informal roles, and are not given as much decision-making power. By providing young PWUD with paid employment opportunities, they can build work experience and be included as equals alongside other paid professionals working in harm reduction—including as paid collaborators in research (e.g. funding applications), decision-makers in policy, and service providers in programs.
6. Digital technologies offer an area of opportunity to communicate and connect with young people. However, for digital technologies to be effective, they will require young PWUD to be involved in the design, implementation, and evaluation of these tools. Young PWUD can and are the ones that most effectively leverage new ways of communication technologies to shift ideologies around the risks and benefits of drugs, including novel and new psychoactive substances. For example, the application “TripApp” was developed by and continues to grow its database through the contributions of young PWUD, showcasing the unparalleled position of young people as they are native to digital solutions and have their finger on the pulse on the realities and trends among their demographic. However, such technology should be available across both developed and developing countries, and more marginalized young people who may not have access to digital devices.
7. To support the above recommendations, adequate funding and resources—and the engagement of youth in the development and implementation of funding—are required. Major funders are yet to provide meaningful and adequate opportunities specifically to support youth engagement and youth-based harm reduction initiatives. Youth-based organizations should be funded directly, with core funding, as autonomous entities so they have the ability and opportunity to act in the best interest of their communities.

## Conclusion

In this commentary, we have highlighted several challenges and areas for improvement in youth engagement in harm reduction. As young PWUD and allies, we recognise that engagement in harm reduction comes with a unique set of challenges, but we believe that institutions could and should prioritize and create capacity for youth engagement and leverage the unique position of young PWUD in the field.

Engagement with young PWUD is critically needed to reduce individual- and population-level health and social outcomes.

Young PWUD are champions in health promotion and harm reduction, both for themselves and the broader community. Their meaningful inclusion and engagement will continue to be a key driver of effective planning, service delivery, and evaluation of harm reduction interventions and programs. However, while the engagement of PWUD is increasingly the norm in harm reduction initiatives, young people are often still an excluded and/or marginalized group in harm reduction contexts. Organizers of both established and emerging harm reduction programs, policies, and research should ensure that initiatives are, if possible, youth-centred—that is, ensuring that youth are meaningfully engaged in all aspects, including governance, programme planning and implementation, evaluation, building partnerships, and communication.

Moving forward, we hope that this commentary serves as a catalyst for young PWUD to take ownership of a coordinated and radical approach, grounded in racial and social justice, whereby youth inclusion is no longer tokenistic but we are given agency and autonomy over decision-making processes, and shape the future of harm reduction, globally. This commentary is a call for more intentional, meaningful, and greater efforts to actively and equitably engage young PWUD in harm reduction. The next generation is depending on it.

## Data Availability

Not applicable.

## Abbreviations

PWUD: People who use drugs
UN: United Nations
HIV: Human immunodeficiency virus
LGBTQI+: Lesbian, gay, bisexual, transgender, intersex, queer/questioning, asexual
